# Comparison of endoscopic balloon dilatation potency using balloons size more or less than 15 mm in the treatment of large bile duct stones: a clinical trial study 

**Published:** 2021

**Authors:** Amir Sadeghi, Arash Dooghaie Moghadam, Shaghayegh Jamshidizade, Mohsen Norouzinia, Negin Jamshidfar, Parna Hosseini

**Affiliations:** 1 *Gastroenterology and Liver Diseases Research Center, Research Institute for Gastroenterology and Liver Diseases, Shahid Beheshti University of Medical Sciences, Tehran, Iran. *; 2 *Basic and Molecular Epidemiology of Gastrointestinal Di* *sorders Research Center, Shahid * *Beheshti University of Medical Sciences, Tehran, Iran*

**Keywords:** Endoscopic balloon dilation, Bile duct stone, Sphincterotomy

## Abstract

**Aim:**

The present study was performed on patients with large bile duct stones to compare clinical outcomes and complications of balloon dilatation treatment between two sizes of balloons, < 15 mm and ≥ 15 mm.

**Background:**

in 1982, the endoscopic papillary balloon dilatation (EPBD) method was presented by Staritz to reduce bleeding and perforation risk of large bile duct stones.

**Methods:**

Patients with large bile duct stones admitted to Taleghani hospital from December 2018 to December 2019 who were the candidates for balloon dilation with limited sphincterotomy. Patients were randomly divided into two groups. In group B, a ≥ 15 mm balloon was used, and in group A, a balloon <15 mm was used. The clinical results of balloon dilation and its complications were recorded and compared.

**Results:**

Most patients had 1 or 2 large bile duct stones, and there was no significant difference in the number of stones. Extraction was successful in 92.8% of group B and 85.7% of group A without significant differences (P = 0.8). Pancreatitis, hemorrhage, cholangitis, and perfusion occurred in 8%, 4.2%, 1.4%, and 2.8% of group B subjects and also in 10%, 2.8%, 0%, and 1.4% of group A subjects, respectively, and the difference between the two groups was not significant.

**Conclusion:**

Generally, this study results showed that balloon size did not have a significant effect on the success rate of bile duct stones. Moreover, considering the lack of significant association between balloon dilatation size and the occurrence of post-endoscopic complications such as pancreatitis, it seems that large-size dilatation has no serious clinical risk.

## Introduction

 In 1974, for the first time, Kawai *et al*. introduced the new method called Endoscopic Sphincterotomy (EST) which gradually became a standard treatment for large bile duct stones removal(1). Although this method was approximately 90% successful to remove large bile duct stones (≥15 mm in diameter), it was associated to an 8 to 12% increase in complications, including pancreatitis, hemorrhage, cholangitis, and perforation (2-4). Thus, in 1982, the endoscopic papillary balloon dilatation (EPBD) method was presented by Staritz to reduce bleeding and perforation risk(5). EPBD, as an alternative technique to EST, was also successfully used in patients with surgical modified anatomy. Moreover, due to using 6 to 10 mm balloons in EPBD, this method can preserve papillary sphincter function in patients with choledocholithiasis and reduced sphincter trauma risks during procedure (2, 6). However, it may be associated to a moderately increased risk of pancreatitis(7).

Due to previous studies, EPBD is effective and safe to remove small and medium bile duct stones, but using this method in the extraction of large gallstones is unfortunately limited because the canal cannot be opened as much as EST in this technique(8). Therefore, there was a need for a new method, until finally, EPBD method using large balloons (12 to 20 mm) was introduced as an alternative technique(9). Since the introduction of EPBD with large balloons, this method was widely used to remove large bile duct stones. This new method probably has the advantages of both sphincterotomy and balloon dilatation. Therefore, due to limited sphincterotomy, it seems to reduce the risk of bleeding and perforating. On the other hand, recent data reported that severe pancreatitis risk is also decreased. Hence, studies have confirmed the safety of EPBD with large balloons, compared to other available methods, and have suggested that this method is effective and safe even without sphincterotomy (10).

 Despite its benefits, balloon dilatation with large balloons (>15 mm) is not accepted by all endoscopists due to concerns about serious complications such as post-endoscopic pancreatitis and bile duct perforation, and choosing the best method remains a challenging issue due to the limitations of previous studies. Moreover, this study is one of the first randomized clinical trials on this subject to the best of our knowledge. Furthermore, the present study was performed to compare balloon dilatation's clinical outcomes and complications using balloons with sizes greater and lower than 15 mm in patients with large bile duct stones. 

## Methods

**Study design**

The current randomized controlled clinical trial study was conducted on patients with large stones (≥15 mm in diameter) who were referred to the endoscopy unit of Taleghani Hospital as a tertiary referral center in Tehran, affiliated with the Shahid Beheshti University of Medical Sciences, Tehran, Iran. The research project was registered in the Iranian Registry of Clinical Trials (IRCT20181117041684N1, 2019/08/13) and also the medical sciences ethics Committee of Shahid Beheshti University of medical sciences (IR.SBMU.MSP.REC.1398.294). All stages of this study were carried out following the ethical principles, rules, and guidelines and in line with the Helsinki Declaration. The patients were informed about the value of their role in this research as well as the procedure and its possible complications, and a consent form was obtained from patients before entering the study.

**Patients**

151 patients consecutively were admitted to our endoscopy unit in Taleghani Hospital from December 2018 to December 2019. Patients in this study include people who have previously been diagnosed with cholelithiasis with large bile duct stones by ultrasound sonography and were referred to our center for treatment of this diagnosis. Large bile duct stones were defined as stones with a diameter of greater than or equal to 15 mm. The patients did not have a significant difference in stone size, and in addition all of them had one or two stones larger than ≥ 15 mm. The exclusion criteria were as follows: 1-pervious sphincterotomy 2-coagulopathy, 3-renal failure, 4-cirrhosis, 5-distal biliary duct stenosis, 6-hepatobiliary malignancy, 7-biliary diverticulosis, 8- pancreatobiliary cancers, 9- segmental stricture or severe angulation of CBD or common hepatic duct (CHD).

Due to using different balloon sizes in the endoscopic dilation and limited spenctortomy, the eligible patients in our study were randomly divided into one of the following two groups: Patients in group A (n=71) experienced dilation with balloon lower than 15 mm and group B (n=80) included patients for whom dilation with greater than or equal 15 mm was performed. Two groups of patients were matched for confounding variables.

**Interventions**

Lidocaine 8% was used as a local anesthetic in patients before the procedure, and scopolamine butyl bromide, pentazocine, and midazolam were intravenously injected. Then, ERCP procedure was started. Endoscopic device (Olympus-tif-q180v-Duodenoscope) was used in our center to perform procedures for all patients. Cannulation is performed with a catheter (MTW Endoskopie), followed by cholangiography to confirm of the presence of CBD stones. Then, with the assist of catheter, guidewire (Boston Scientific) inserted. Limited EST is then performed to prevent perforation and pancreatitis. Afterward, depending on which of the two groups A or B belongs to the patient, Hurricane RX Biliary Balloon Catheter (Boston Scientific) for group A and CRE RX Biliary Balloon Dilatation Catheters (Boston Scientific) for group B were used. The balloons were filled with sterile water to their maximum defined size, and after approximately 30 seconds, the balloons were quickly emptied. All patients were hospitalized and monitored for 24 hours after the procedure.

**Statistical analysis**

Patients’ Demographic characters, clinical data, outcomes, and complications of the procedures were recorded and compared between the two groups. We used the Kolmogorov-Smirnov test for normality. Chi-square, independent sample student t-test, and Fisher’s exact test were used as analytical statistics. A P <0.05 was considered statistically significant. The statistical analyses were performed using the statistical software package SPSS 20 for Windows (SPSS, Inc., Chicago, IL). 

## Results

**Description of the study**

151 patients were allocated into two groups, including group A (balloon size < 15 mm, n=71) and group B (balloon size ≥ 15 mm, n=80). The patients’ means of age in A & B groups were 74.3 and 71.8 years, respectively. Regarding gender, females constituted 53.4% and 51%, and males comprised 46.6% and 49% in groups A and B, respectively. The patients’ means of height and weight have been shown in Table 1. 

There were no significant differences between the two groups regarding the prevalence of malignancy, hypertension, chronic kidney disease, end-stage renal disease (ESRD), chronic obstructive pulmonary disease (COPD), asthma, ischemic heart disease, and congestive heart failure. 

**Table 1:** Height and weight of patients in the studied groups

Variables	Study Group	Mean ±SD	P value
Height (cm)	B	166.6±14.2	0.3
	A	163.4±13.8	
Weight (kg)	B	71.2±6.8	0.2
	A	75.6±6.8	

The results of laboratory parameters (serum levels of alkaline phosphatase, total bilirubin, direct bilirubin, amylase, and lipase) in the patients have been shown in Table 2.

**Table 2:** Serum levels of laboratory parameters of hepatic function

Variables	Groups	n	Mean±SD	P value
Alkaline phosphatase (mg/dl)	B	73	335±57.4	0.9
	A	67	387±57.4	
Total Bilirubin (mg/dl)	B	73	2.29±0.49	0.5
	A	67	2.89±0.89	
Direct bilirubin (mg/dl)	B	73	2.6±0.85	0.01*
	A	67	1.2±0.3	
Amylase (mg/dl)	B	72	76.5±24.9	0.003*
	A	67	82.4±32.6	
Lipase (mg/dl)	B	72	70.8±34.5	0.006*
	A	67	73.6±37.8	

*statistically significant

In B & A groups, 70 (88%) and 56 (78%) of patients had Large bile stones, respectively (Table 3). Most of the patients in both groups had either 1 or 2 large bile duct stones (P-value = 0.1).

**Table 3:** The number of large bile duct stones in patients in the two studied groups

Groups	Number of stones	n	%
B	1 or 2	49	70
	3 or 4	10	14
	≥5	11	16
A	1 or 2	39	70
	3 or 4	10	18
	≥5	7	12

***Comparison of stone removal success***

Due to our results, large bile duct stones were successfully removed at the first attempt in 92.8% of the patients in group B (balloon size ≥15 mm) and 85.7% of those in group A (balloon size < 15 mm). The difference between the two groups was not statistically significant (P-value = 0.8).

***Comparison of Complications***

Due to our results, post-endoscopic pancreatitis was detected in 8% and 10% of patients in the groups with balloon sizes of ≥ 15 mm and <15 mm, respectively (P-value = 0.9). None of the patients showed severe pancreatitis in neither of the groups Cholangitis occurred in 1 (1.4%) patient in the group with dilatation balloon size ≥ 15 mm while no one showed this complication in group A (P-value = 0.4). Furthermore, post-endoscopy bleeding was identified in 1 (1.4%) and 3 (4.2%) patients in group A (balloon size <15 mm) and group B (balloon size >15 mm), respectively. Perforation occurred in 2 patients in group B and in 1 patient in group A. There were no significant differences in the events of cholangitis, bleeding, perforation, and mortality between the two groups (Table 6). Among three patients who had a hemorrhage in group B, one of them had severe requiring surgery. Finally, one patient died in group B while no death was recorded in group A. The difference between the two groups was not statistically significant.

**Table 4:** Comparison of complications after endoscopy between the two studied groups

Study groups	B (balloon size ≥ 15 mm)	A (balloon size < 15 mm)	P-value
Pancreatitis	6 (8)^*^	5 (10)	0.9
Cholangitis	1 (1.4)	0 (0)	0.4
Bleeding	3 (4.2)	1 (1.4)	0.2
Perforation	2 (2.8)	1 (1.4)	0.1
Mortality	1 (1.4)	0 (0)	0.3

* N (%)

## Discussion

In this study, our main objectives were to determine the relationships between balloon size and the stone removal's success rate and post-endoscopy complications. 

The EPBLD is a convenient method to remove large bile duct stone which is increasingly used in ERCP referral centers (11). Previous studies reported that if the mechanical lithotripsy is not used, this method can be effective more than 74% (8, 12-16). The success rate in EPLBD with EST is 96.5%, while in EPBD, this success is lower which reported about 90%, and the meta-analysis already showed that the overall success rate in EPLBD with EST is usually higher than EPBD (17). These reports are similar with our research findings that the technical success in the group which used a balloon with a size more than 15 mm which is 92.8%; also, the success rate in EPLBD group was higher in our study. However, there was no statistically significant difference between the two groups in the results of the study of successful removal of large bile duct stone, indicate that the balloon size had no impact on the success rate of large bile duct stones removal.

The complication rate is another important issue which is compared between the two groups in our article. The complication rate in cases who EPLBD has been performed with EST has been reported in the range of 0-17%. Complications reported in previous articles were mostly moderate or mild and improved with conservative treatments (17). However, severe complications, such as death, were previously reported (18-20). In metanalysis, the most common complication in cases who EPLBD was performed with EST was bleeding, which was about 3.6%, followed by pancreatitis with about 2.6% (17). However, our findings indicate a higher prevalence of pancreatitis. Pancreatitis in ERCP is a common complication that multifactorial causes, such as mechanical causes and trauma, are among the most important factors during various procedures. Trauma to the papilla during cannulation causes Oddi spasm and edema which block the pancreas' normal flow (21). Therefore, due to the involvement of many factors, it seems difficult to investigate pancreatitis causes in different groups. It may be assumed that the dilatation balloon, given that the radical force exerted by the balloon, which is responsible for dilation, enters far away from the pancreatic duct, and the orifice is less damaged and less pancreatitis occurs. Moreover, because EPLBD reduces papilla trauma and requires less mechanical force lithotripsy and forceps stone extraction, less pancreatitis is expected than in EPBD(22). Finally, however, there was no statistically significant difference between the two groups in which different balloon sizes were used in our study. Bleeding is another common complication (17)which was studied in each group and did not differ significantly. Various factors have previously been considered factors involved in ERCP bleeding, including coagulopathy and low platelets, improper positioning, incorrect electrocautery and Long cuts known as zippers (23, 24). Park et al. showed that cirrhosis, stone size greater than 16 mm, and full ESD were independent factors involved in the bleeding event in EPLBD, and EPLBDs performed with large ESD was also shown to have increased bleeding (19). Regardless of the two complications mentioned, in EPLBDs performed with EST, the most serious complication is a perforation which in some reports has caused death through the septic shock, and in these cases, protocols and guidelines should be carefully followed (16, 17, 19, 25). In our studies, there were no significant differences among other complications such as perforation between the two groups of EPLBD and EPBD; so, it can be generally concluded that using both methods can be a safe method for patients. However, our study had several limitations which should be considered in analyzing these results. These limitations are as follows: firstly, our study is a single center, and our population study is limited; therefore, it is better to design a multicentre study to eliminate these two limitations. Moreover, we do not adjust and entered other probable efficient factors such as the duration of our procedures or the method used to remove large stones after dilation. On the other hand, in our study, we followed all of our patients only for 24 hours. Hence, there is no evidence and data about long-term complications and also the probability of stone recurrence. Future studies with long- term follow up should be designed.

In general, the results of the present study showed that the balloon size (greater or less than 15 mm) had no significant impact on the success rate of large bile duct stones removal in patients undergoing EPBD. Regarding the lack of a significant association between dilatation balloon size and the occurrence of post-endoscopic complications such as pancreatitis, cholangitis, bleeding, and profusion, this method seems to provide a safe approach with minimal clinical ris.

## Conflict of interests

The authors declare that they have no conflict of interest.

## References

[1] Kawai K, Akasaka Y, Murakami K, Tada M, Koli Y (1974). Endoscopic sphincterotomy of the ampulla of Vater. Gastrointest Endosc.

[2] Stefanidis G, Christodoulou C, Manolakopoulos S, Chuttani R (2012). Endoscopic extraction of large common bile duct stones: A review article. World J Gastrointest Endosc.

[3] Gupta N, Poreddy V, Al-Kawas F (2008). Endoscopy in the management of choledocholithiasis. Curr Gastroenterol Rep.

[4] Rouquette O, Bommelaer G, Abergel A, Poincloux L (2014). Large balloon dilation post endoscopic sphincterotomy in removal of difficult common bile duct stones: a literature review. World J Gastroenterol.

[5] Staritz M, Ewe K, Meyer zum Büschenfelde KH (1982). Endoscopic papillary dilatation, a possible alternative to endoscopic papillotomy. Lancet.

[6] Rostami Nejad M, Rostami K, Yamaoka Y, Mashayekhi R, Molaei M, Dabiri H (2011). Clinical and histological presentation of Helicobacter pylori and gluten related gastroenteropathy. Arch Iran Med.

[7] Weinberg BM, Shindy W, Lo S (2006). Endoscopic balloon sphincter dilation (sphincteroplasty) versus sphincterotomy for common bile duct stones. Cochrane Database Syst Rev.

[8] Ersoz G, Tekesin O, Ozutemiz AO, Gunsar F (2003). Biliary sphincterotomy plus dilation with a large balloon for bile duct stones that are difficult to extract. Gastrointest Endosc..

[9] Meine GC, Baron TH (2011). Endoscopic papillary large-balloon dilation combined with endoscopic biliary sphincterotomy for the removal of bile duct stones (with video). Gastrointest Endosc.

[10] Shim CS, Kim JW, Lee TY, Cheon YK (2016). Is endoscopic papillary large balloon dilation safe for treating large CBD stones?. Saudi J Gastroenterol.

[11] Fujisawa T, Kagawa K, Hisatomi K, Kubota K, Nakajima A, Matsuhashi N (2014). Endoscopic papillary large-balloon dilation versus endoscopic papillary regular-balloon dilation for removal of large bile-duct stones. J Hepatobiliary Pancreat Sci.

[12] Bang S, Kim MH, Park JY, Park SW, Song SY, Chung JB (2006). Endoscopic papillary balloon dilation with large balloon after limited sphincterotomy for retrieval of choledocholithiasis. Yonsei Med J.

[13] Heo JH, Kang DH, Jung HJ, Kwon DS, An JK, Kim BS (2007). Endoscopic sphincterotomy plus large-balloon dilation versus endoscopic sphincterotomy for removal of bile-duct stones. Gastrointest Endosc.

[14] Maydeo A, Bhandari S (2007). Balloon sphincteroplasty for removing difficult bile duct stones. Endoscopy.

[15] Minami A, Hirose S, Nomoto T, Hayakawa S (2007). Small sphincterotomy combined with papillary dilation with large balloon permits retrieval of large stones without mechanical lithotripsy. World J Gastroenterol.

[16] Attasaranya S, Cheon YK, Vittal H, Howell DA, Wakelin DE, Cunningham JT (2008). Large-diameter biliary orifice balloon dilation to aid in endoscopic bile duct stone removal: a multicenter series. Gastrointest Endosc.

[17] Kim JH, Yang MJ, Hwang JC, Yoo BM (2013). Endoscopic papillary large balloon dilation for the removal of bile duct stones. World J Gastroenterol.

[18] Ghazanfar S, Qureshi S, Leghari A, Taj MA, Niaz SK, Quraishy MS (2010). Endoscopic balloon sphincteroplasty as an adjunct to endoscopic sphincterotomy in removing large and difficult bile duct stones. J Pak Med Assoc.

[19] Park SJ, Kim JH, Hwang JC, Kim HG, Lee DH, Jeong S (2013). Factors predictive of adverse events following endoscopic papillary large balloon dilation: results from a multicenter series. Dig Dis Sci.

[20] Paspatis GA, Konstantinidis K, Tribonias G, Voudoukis E, Tavernaraki A, Theodoropoulou A (2013). Sixty- versus thirty-seconds papillary balloon dilation after sphincterotomy for the treatment of large bile duct stones: a randomized controlled trial. Dig Liver Dis.

[21] Tryliskyy Y, Bryce GJ (2018). Post-ERCP pancreatitis: Pathophysiology, early identification and risk stratification. Adv Clin Exp Med.

[22] Youn YH, Lim HC, Jahng JH, Jang SI, You JH, Park JS (2011). The increase in balloon size to over 15 mm does not affect the development of pancreatitis after endoscopic papillary large balloon dilatation for bile duct stone removal. Dig Dis Sci.

[23] Khoshbaten M, Rostami Nejad M, Farzady L, Sharifi N, Hashemi SH, Rostami K (2011). Fertility disorder associated with celiac disease in males and females: fact or fiction?. J Obstet Gynaecol Res.

[24] Ratani RS, Mills TN, Ainley CC, Swain CP (1999). Electrophysical factors influencing endoscopic sphincterotomy. Gastrointest Endosc.

[25] Hwang JC, Kim JH, Lim SG, Kim SS, Shin SJ, Lee KM (2013). Endoscopic large-balloon dilation alone versus endoscopic sphincterotomy plus large-balloon dilation for the treatment of large bile duct stones. BMC Gastroenterol.

